# Prediction Mortality Rate Due to the Road-Traffic Accidents in Kazakhstan

**Published:** 2020-01

**Authors:** Nurbek IGISSINOV, Alma AUBAKIROVA, Galiya ORAZOVA, Gulnur AKPOLATOVA, Saltanat URAZOVA, Dinar TARZHANOVA, Akmaral ZHANTUREYEVA, Yerlan KUANDYKOV

**Affiliations:** 1 Department of Surgical Diseases № 2, Astana Medical University, Nur-Sultan, Kazakhstan; 2 Department of Science and Analytic, International High School of Medicine, Bishkek, Kyrgyzstan; 3 Central Asian Cancer Institute, Nur-Sultan, Kazakhstan; 4 Eurasian Institute for Cancer Research, Bishkek, Kyrgyzstan; 5 Republican Center for Health Development, Nur-Sultan, Kazakhstan; 6 Department of Public Health, Astana Medical University, Nur-Sultan, Kazakhstan; 7 Department of General and Clinical Pharmacology, Astana Medical University, Nur-Sultan, Kazakhstan; 8 Department of General Medical Practice №2, Astana Medical University, Nur-Sultan, Kazakhstan; 9 Department of Family Medicine, South Kazakhstan Medical Academy, Shymkent, Kazakhstan

**Keywords:** Road-traffic accidents, Mortality, Prediction, Kazakhstan

## Abstract

**Background:**

As a result of the road traffic accidents 1.25 mln. of working-age people die each year on the roads. Frequency of the RTA is 11 times higher in our country than in Europe, that influence on demographic and economic situation in the republic. Creation of the math modeling and prediction of traffic mortality rate in Kazakhstan will allow to develop measure on its decrease.

**Methods:**

Short-term dotted prediction of population mortality level of Kazakhstan was used, in particular – methods of regressive analysis. General prognosis throughout the country up to 2021 was made on the basis of data for 1999–2018. The more relevant method for prediction is exponential function taking into account the features of mortality rate level trend.

**Results:**

Prediction of traffic fatalities without division into the age-related groups for 2019 is 2132±181 case with a probability 2/3. Expected levels for 2020–2027 cases, for 2021–1927 cases.

Annual mortality decrease rate according to the 0–19 age-related at an average is 6.4% among men and 5.8% among women, according to age group as a whole – by 6.2%; from 20 up to 64 age related group – 5.1 % on all population category; older 65 age –group is by 2.2 %, 3.7 % among men, 2.9% among women as a whole.

**Conclusion:**

In the foreseeable future the number of traffic deaths in Kazakhstan will tend to decrease at a slower pace. Mortality rates due to road traffic accidents among working-age men will be 3 times higher than women in this age group.

## Introduction

At the present time problems due to the road traffic accidents (RTA) relate to the most important issues in healthcare and social policy in all countries of the world. Approximately 1,25 mln. of working age people die each year on the roads (1).

According to the WHO and the World Bank by 2020 the deaths as a result of the RTA will have become one of the main reasons of mortality in many countries of the world (2). In the projected rise of the road accidents world ranking as a main reason of population deaths the important role plays developing countries whereas in developed countries the road traffic accidents deaths are projected to decrease by 30% by 2020 (3).

The mortality as a result of RTA is a one of the most serious problems of healthcare of Kazakh-stan. Increase of population number and improvement of economic situation have led to the rise of RTA number in the country. Today in Kazakhstan the mortality due to RTA is 30.6 per 100ths. of population at an average. Our country takes first place in European region of the WHO by a significant margin (4). Such human losses are comparable with losses in major military conflicts.

In order to enhance control under the situation in 2008 Kazakhstan adopted a law within the framework of which administrative responsibility was strengthened for nonuse of safety belts, entering an oncoming lane, driving under the influence of drink, etc. (5). Nevertheless, frequency of the RTA is 11 times higher in our country than in Europe (6, 7), that influences on demographic and economic situation in the republic.

Increase in income among the population that has been observing over the last few years certainly will lead to a mass nation and region motorization. As a fact, it can lead to a higher level of mortality due to RTA in the country (8).

Research on mortality level prediction due to RTA has not been conducted previously as one of the key factors of working-age population death. Prediction level modeling among the country population will play an important role in planning and managing measures on improvement of road traffic safety. We aimed to provide mathematical and predictive modeling of mortality due to road traffic accidents in Kazakhstan.

## Materials and Methods

Short-term dotted prediction of population mortality level of Kazakhstan as a result of the RTA was carried out on the basis of the facts on RTA mortality level for 1999–2018. Exponential function via time-series modeling of mortality level changing as a much more effective method of mortality modeling was used in the research (9–11).

Our research includes three stages: studying population mortality over the period under review, modeling and prediction of mortality up to 2021 on the basis of obtained models.

First stage – studying of mortality due to RTA in Kazakhstan for 1999–2018. We determined general (crude) markers of mortality per 100 ths. of the population for the period under review based on information about mortality rate due to RTA official registration, data of the Statistical Agency on age-sex composition population number, official statistical data of the Ministry of Health of the Republic of Kazakhstan. The dynamics of traffic fatalities was studied in three age groups: first group – up to 19 yr old, second one – from 20 up to 64 yr old and the third one – older 65 yr old. Breakdown of abovementioned age groups was made according to participation activity criteria of certain age groups of population in the road traffic (12).

Second stage – math modeling of mortality rate due to the RTA in Kazakhstan over the period under review. Selection of a proper model was made into consideration with process content and peculiarities of traffic fatalities dynamics in three abovementioned age groups.

Methods of correlation analysis and time-series models that had been built on the basis of series of dynamics over the period and its extrapolation were used for calculations (10, 11, 13).

We chose the exponential function of the following form to be the most appropriate to reflect the specifics of the examined process.

$$Y_{t}=ce^{\mathrm{bt}}u_{t}$$

where ***c*** and ***b***- parameters of the model, defining its shape;
***t***- factor variable (time, observation sequence number);
***e*** - base of natural logarithms;
***u_t_*** - malfunction.

In an exponential model parameter *b* represents the relative size (percentage) of changes in the level of the indicator; this parameter is assumed to be constant. The sign of the parameter depends on the nature of the series dynamics; in our case parameter *b* carries a negative sign as the dynamics of road traffic accident mortality rate in the given period tends to decrease. Hence, as the process evolves the absolute value of mortality rate will decrease.

Coefficient of determination in all cases was within the scope of from 0.83 up to 0.92. Testing of models and regression coefficients at a level of value 0.01 once again in all cases shoed its reliability. The facts show that used as a basis a simple linear model extremely prompt apprizes the series trend. Calculated models are shown in Table 1 in more conventional form.

**Table 1:** Predictive models

***Object***	***Period of monitoring***	
	***1999–2007***	***2008–2018***
Males	*Y_t_*=801+292*t*+*ɛ*, *R*^2^=0.86	*Y_t_*=2783−105*t*+*ɛ*, *R*^2^=0.90
Females	*Y_t_*=218+111*t*+*ɛ*, *R*^2^=0.83	*Y_t_*=1009−36*t*+*ɛ*, *R*^2^=0.85
Population at large	*Y_t_*=1018+403*t*+*ɛ*, *R*^2^=0.86	*Y_t_*=3798−144*t*+*ɛ*, *R*^2^=0.92

Third stage – prediction. Further, we determined a mortality rate predictive level due to the RTA in Kazakhstan via extrapolation of obtained models. Unfortunately, a comparatively short database allowed to extrapolate the obtained models only 2–3 years in advance, in our case until 2021.

The whole statistical analysis was carried out using of SAS and Stata 12 software packages.

This article is a fragment of the doctoral thesis paper by Alma Aubakirova.

## Results

### Studying of mortality rate markers due to the RTA in Kazakhstan

We have studied crude markers of mortality due to the RTA in Kazakhstan over the period 1999–2018 (Table 2).

**Table 2:** General (crude) markers of Kazakhstan population mortality due to road traffic accidents per 100,000 population according to age categories, 1999–2018

***Age***	***1999***	***2000***	***2001***	***2002***	***2003***	***2004***	***2005***	***2006***	***2007***	***2008***	***2009***	***2010***	***2011***	***2012***	***2013***	***2014***	***2015***	***2016***	***2017***	***2018***
0–4	4.9	5.2	4.3	4.1	5.3	5.6	6.7	8.4	6.9	5.8	6.2	6.0	5.9	5.8	5.8	5.2	4.1	4.3	3.1	4.0
5–9	5.3	6.1	6.5	5.9	6.8	9.0	8.7	10.8	11.5	9.6	5.8	8.4	5.5	6.4	5.6	5.1	4.2	4.0	3.9	3.7
10–14	4.4	3.3	3.8	3.6	3.4	5.4	5.7	8.3	9.3	6.0	5.0	5.5	5.9	5.2	4.1	4.1	5.0	4.2	3.5	3.2
15–19	7.6	8.7	10.4	8.6	11.3	12.9	16.3	18.9	23.2	15.7	12.3	14.3	13.0	12.4	11.9	10.1	8.7	11.1	7.6	7.4
20–24	15.4	17.0	21.5	19.2	19.9	26.7	35.7	45.9	43.6	34.1	26.6	24.1	26.4	23.9	25.3	21.6	19.3	18.4	16.0	15.5
25–29	21.4	19.2	21.7	22.4	24.1	30.8	40.1	50.5	52.0	36.9	30.7	30.1	24.9	28.1	26.9	21.8	18.8	18.5	14.8	15.7
30–34	21.6	21.5	22.1	23.2	23.7	30.4	35.0	49.7	55.3	36.3	28.6	29.2	28.6	26.0	27.2	23.1	20.0	20.2	17.7	13.2
35–39	21.5	21.1	21.6	22.3	24.3	32.7	37.1	48.2	45.9	33.4	30.3	29.6	25.4	26.0	25.5	21.9	19.7	19.5	16.4	16.6
40–44	21.4	18.9	21.9	21.3	23.5	28.3	35.8	42.8	42.6	32.8	29.5	30.3	27.5	24.4	23.0	22.6	19.9	19.1	18.2	18.0
45–49	17.6	17.0	19.9	17.5	21.4	22.7	31.2	45.1	38.9	28.5	27.0	27.3	26.5	25.1	26.6	24.3	19.6	21.8	17.9	17.1
50–54	17.6	18.9	18.3	19.7	20.9	24.4	32.1	37.6	34.0	27.8	29.0	25.6	24.1	27.4	27.0	21.5	20.3	21.7	15.8	17.6
55–59	12.2	14.8	14.6	16.7	19.6	26.6	29.1	34.4	35.6	24.3	24.3	27.6	24.4	26.8	25.4	19.1	20.5	23.1	16.0	19.1
60–64	15.0	12.9	15.5	16.5	15.6	20.2	22.2	36.7	27.7	24.9	20.0	26.9	25.1	20.8	24.5	18.2	19.4	17.5	17.7	17.4
65–69	11.2	13.6	17.2	14.3	17.7	19.6	25.1	25.8	24.5	20.0	22.1	21.4	19.1	19.5	21.4	20.9	14.1	20.6	15.2	12.3
70–74	12.7	17.5	12.3	15.6	15.2	13.2	23.3	26.3	25.3	21.1	19.9	21.8	21.7	18.4	20.4	11.9	12.8	17.3	15.4	13.4
75–79	16.8	11.9	12.3	14.1	17.9	23.7	21.0	24.8	26.0	21.3	22.9	19.4	19.4	17.7	23.4	15.6	17.4	14.7	14.4	12.1
80–84	13.4	17.2	16.9	13.0	8.5	20.7	22.9	28.7	20.9	20.3	15.3	15.2	15.3	12.5	11.1	13.1	15.1	18.0	11.7	17.3
85+	14.5	4.8	13.5	12.4	5.7	5.8	20.1	15.8	10.0	7.7	10.9	6.6	11.0	13.1	9.6	10.1	12.3	4.5	11.9	8.1
Total	13.4	13.4	14.8	14.6	16.1	20.1	25.0	32.0	31.8	23.6	20.6	20.9	19.5	19.1	19.1	16.2	14.5	14.9	12.3	12.0

A relative stability of process operation conditions has been violated. Over the period from 1999 till 2007 the level of traffic mortality at an average increased by 2.36 cases per 100ths of population of the country. Beginning from 2008 the process reversed course: annually at an average the number of traffic losses became decrease by 1.10 cases per 100ths people. As was mentioned in the “Materials and methods” chapter, the highest markers of mortality were determined in age group from 20 up to 64 yr old.

### Math modeling of time-series mortality level changes

Time-series modeling of the marker under review throughout the research period from 1999 till 2018 shows that the series trend on traffic mortality level is approximated in the best possible way by second-degree polynomial, i.e. an equation *Y_t_* = *b*_0_ + *b*_1_*t* + *b*_3_*t*^2^. What is more such affirmation is specifically for the series of all sexage groups and population as a whole. Table 3 shows the obtained models of relatively male and female parts of population as a whole with breakdown according to their ages: up to 19 yr old, from 20 till 64 yr old, 65 yr old and older.

**Table 3:** Approximation of mortality rate dynamics due to the road traffic accidents in Kazakhstan, 1999–2018

***№***	***Age group (yr)***	***Male population***	***Female population***	***Population at large***
1	0–19	y=−1.8045x^2^+34.24x+154.87	y=−0.9425x^2^+20.216x+73.677	y=−2.853x^2^+55.771x+225.92
		R^2^=0.58	R^2^=0.46	R^2^=0.61
2	20–64	y=−13.789x^2^+293.16x+734.57	y=−4.4903x^2^+98.72x+148.14	y=−18.28x^2^+391.88x+882.71
		R^2^=0.63	R^2^=0.63	R^2^=0.63
3	65 and older	y=−0.5681x^2^+12.723x+58.38	y=−0.5987x^2^+13.041x+40.731	y=−1.1668x^2^+25.764x+99.111
		R^2^=0.53	R^2^=0.54	R^2^=0.56

**Fig. 1:**
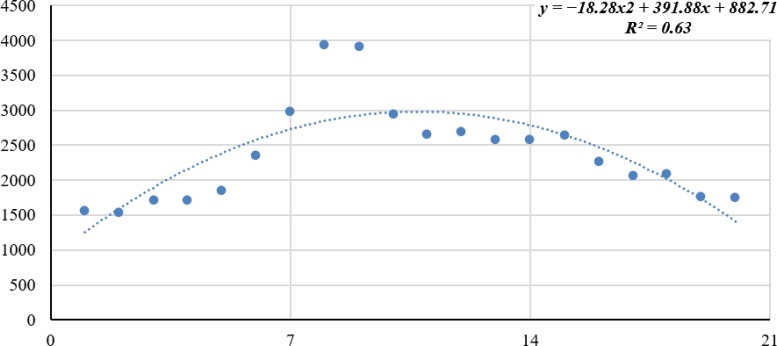
Dynamics of mortality from traffic accidents among the population of Kazakhstan in the age category from 20 to 64 years, 1999–2018

Figure 1 represents the trend of traffic fatalities among the population of Kazakhstan. For more effective and illustrative examples, we demonstrated the mortality trend in 20–64 age group.

Determination coefficients of the obtained approximations are quite significant. However, given equations (second-degree polynomial) cannot be used in prediction of a phenomenon under review. This is due to the fact that the main condition for extrapolation is violated in the process under the research, which is a relatively stability of process development. That is why the methodically correct formulation and modeling of the process means using of data beginning from 2008 for analysis and prediction.

Although, a lot of processes are enough simply approximated with using linear models (as it is shown above), in reality social processes and phenomenon have nonlinear feature and respectively they suppose attraction of nonlinear functions for its approximation as possible.

**Fig. 2:**
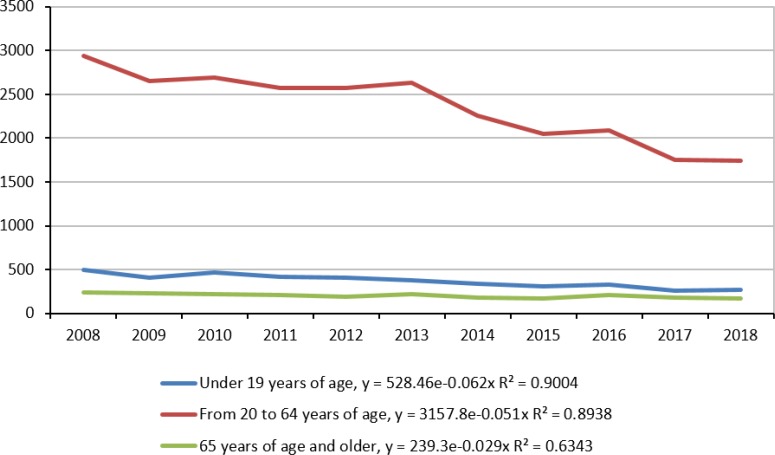
Exponential function approximation of the traffic accident mortality cases in Kazakhstan across three age groups, 2008–2018

Table 4 shows parameters of exponential functions reflecting traffic fatality trend during the period from 2008 till 2018. The most important information has coefficients at factor variable ***t***. Figure 2 represents the approximation of the exponential function process.

**Table 4:** Nonlinear models of the road traffic accident mortality cases in Kazakhstan by the age groups

***№***	***Age group (yr)***	***Male***	***Female***	***Both genders***
1	0–19	y=329.88e^−0.064t^	y=198.19e^−0.058t^	y=528.46e^−0.062t^
		R^2^=0.89	R^2^=0.84	R^2^=0.90
2	20–64	y=2423e^−0.051t^	y=734.48e^−0.051t^	y=3157.8e^−0.051t^
		R^2^=0.88	R^2^=0.87	R^2^=0.89
3	65 and older	y=126.16e^−0.022t^	y=113.05e^−0.037t^	y=239.3e^−0.029t^
		R^2^=0.33	R^2^=0.74	R^2^=0.63

### Traffic fatality level prediction in Kazakhstan

Prognosis of mortality level in Kazakhstan for 2019–2021 is made on the basis of obtained models. For more precise visualization of obtained results, we represented the data in absolute numbers. In Table 5 possible deviations from expected level correspond to sizes of relevant standard errors of models.

**Table 5:** The expected number of road traffic accident mortality cases in Kazakhstan for 2019

***№***	***Age group (yr)***	***Male***	***Female***	***Both genders***
1	0–19	153±18*	99±15*	251±29*
2	20–64	1314±125*	398±42*	1712±155*
3	65 and older	97±13*	73±7*	169±16*

* with a probability of 2/3 (standard error)

Prediction capability for medium and long-term perspectives are limited by the fact that models are made on the basis of relatively short-time investigation database. In other words, the further the prognosis horizon is the less reliable the expected outcomes are. Trend extrapolation on the basis of models trend up to 2020 and 2021 gives single-value prediction presented in Table 6.

Prediction of a general level of traffic fatalities without division into the age groups for 2019 is 2127±181 case with a probability of 2/3. Expected levels of the marker for 2020 is 2022 cases, for 2021–1921 case.

**Table 6:** The expected number of road traffic accident mortality cases in Kazakhstan for 2020–2021

***№***	***Age group (yr)***	***Male***	***Female***	***Both genders***
2020				
1	0–19	144	93	236
2	20–64	1249	378	1627
3	65 and older	95	70	164
2021				
1	0–19	135	88	222
2	20–64	1186	360	1546
3	65 and older	93	67	159

## Discussion

The number of traffic fatality cases in Kazakhstan has a tendency for reduction with delayed decrease rate. According to the WHO prognosis, the same tendency is expected to be in many countries of the world (4). In spite of prognosis about annual reduction of number of traffic fatality, the size of the reduction will reduce every year.

The entry into force in 2008 of the new Law on Administrative Violations and the system of fines and other penalties for driving violations in Kazakhstan (6) radically changed the stricter conditions for moving entities by cars, affecting the increase responsibility of the population on the roads. This is one of the key factors of the “Haddon’s Matrix” model, which affects the degree of road accidents (14). According to this model, further sustainable reduction in mortality rate due to the RTA in Kazakhstan can be facilitated by improving the quality of roads and optimizing the operation of emergency medical services. That is why there is a need to further improve the state policy of the country to reduce the mortality rate of citizens due to the RTA.

When modeling the number of traffic fatalities in Kazakhstan we had problems associated with choosing the most suitable function. As a model for prediction, in principle, different functions could be used, such as autoregression, autoregressive moving average (ARMA), autoregressive integrated moving average (ARIMA), seasonal autoregressive moving average (SARIMA), as well as Li-Carter and etc. (15, 16). There are works on prediction of various indicators in healthcare using various models that have sufficient predictive power and reliability, where the logarithmic function was used (17–19). However, here we should take into account the nature of the phenomenon under study, as in our case. Therefore, the prediction of a specific phenomenon in a particular country should be approached strictly individually.

The available literature also contains interesting works by various authors who simulated a short-term prediction of traffic fatalities. Among the works using various models for predicting mortality due to the RTA, most often, there are the most recent manuscripts of Iranian scientists. Further, we decided to conduct a brief review of the scientific papers that interested us.

The ARIMA model was used to predict the seasonality of accidents on the roads of Taybad (20). The study analyzed data on mortality due to road accidents for 5 years (2007–2012). However, the use of the ARIMA method did not allow scientists to identify the expected seasonality of the studied phenomenon, which once again indicates the difficulty of selecting the appropriate function to conduct a more accurate prediction of the consequences of accidents.

A group of Iranian scientists conducted a short-term prediction of road traffic deaths in the Zanjan province of Iran using time series modeling (21). To remove instability from the time series, the authors used several models at once (ARMA, ARIMA, SARIMA). This combination of prediction models allowed us to establish downward trend mortality in the study area for the next 4 years. According to the authors, the identified positive dynamics was achieved as a result of an effective government policy to introduce some new regulatory acts aimed at reducing the mortality of the Iranian population due to the RTA.

The seasonality of mortality rate due to the RTA was determined in 2016 (22). When applying SARIMA based on a three-year prognosis of time series (2013–2015), the lowest mortality rates due to the RTA are observed in the spring and autumn months. Earlier in 2015 a team of scientists from China and the USA also studied the seasonality of traffic fatalities in China using the SARI-MA model (23). Analysis of data for 2000–2011 using this model allowed scientists to determine the exact monthly prediction of mortality due to the RTA for one year in advance. Thus, the abovementioned two independent groups of scientists proved the effectiveness of the math model of SARIMA prognosis, which allows conducting a detailed prediction of the mortality rate due to the RTA depending on the time of year.

Taking into account the difficult meteorological conditions in the winter and the geographical features of Kazakhstan the SARIMA predicting model can be successfully applied in our future work a few years after the accumulation of the mortality database depending on the time of year. Unfortunately, seasonal-like statistics on road traffic accidents in Kazakhstan is incomplete, which in turn complicates our task.

During the literary search, we were unable to find the results of studies by other authors on predicting mortality rate due to the RTA using ETS (exponential smoothing). This only emphasizes the originality of our work. We believe that we were able to conduct an effective short-term prediction of mortality from the phenomenon under study with a fairly high probability (2/3).

Thus, the strength of our research is that despite the difficulties identified during the study associated with the instability of the studied phenomenon we have identified the most suitable method for predicting (non-linear approximation functions) mortality due to the RTA for the coming years. This method can be further used to predict other causes of mortality occurring in a similar scenario.

The weakness of the study is the short-term prediction, associated with a relatively short-time database (11 years) of observations.

Longer-term prognosis can provide modified predicting models related to per capita income. A prediction model that depends on the level of income of the population makes it possible to determine later and higher peaks of mortality rate due to the RTA than the verified time-dependent model. Mortality from the studied cause in the world will begin to decline only after the global peak (up to 1.8 million deaths) in 2035. At the same time, the lower the country's income per capita is, the later mortality due to the RTA is (8).

## Conclusion

In the foreseeable future, the number of traffic deaths in Kazakhstan will tend to decrease at a slower pace. Mortality rates due to road traffic accidents among working-age men will be 3 times higher than women in this age group.

The radical turn in the dynamics of mortality among the population of the Republic of Kazakhstan as a result of the road traffic accidents in the direction of decreasing lethal outcomes is explained, first of all, by cardinal changes in the paradigm of driver behavior on the roads. The main reason for this change is the legislative initiatives undertaken in 2008 aimed at increasing the responsibility (first of all, material and financial) of the participants in the movement, especially motorists.

## Ethical considerations

Ethical issues (Including plagiarism, informed consent, misconduct, data fabrication and/or falsification, double publication and/or submission, redundancy, etc.) have been completely observed by the authors.
